# Experiences of Health Care Access Among Trans Adolescents in the Region of Murcia, Spain

**DOI:** 10.3390/healthcare13222953

**Published:** 2025-11-18

**Authors:** María del Mar Pastor-Bravo, María Marín-Rodríguez, David Martín-Castillo, José Antonio Jiménez-Barbero, José Joaquín García-Arenas, María Sánchez-Muñoz

**Affiliations:** 1 Faculty of Nursing, University of Murcia, 30120 Murcia, Spain; 2 Biomedical Research Institute of Murcia Pascual Parrilla–IMIB, 30120 Murcia, Spain; 3 Occupational Therapy Department, Catholic University of San Antonio de Murcia, 30107 Murcia, Spain; 4 Mental Health Centre of Cartagena, Murcia Health Service, 30201 Cartagena, Spain

**Keywords:** transgender, adolescent, youth, health service, gender identity

## Abstract

**Objective:** The objective of this research is to explore the healthcare access experiences of trans and gender-diverse adolescents in the Region of Murcia (Spain). **Methodology:** An exploratory qualitative study was conducted using an interpretative phenomenological approach, consisting of semi-structured interviews with adolescents and their families. The study sample consisted of 20 participants: 10 trans and gender-diverse adolescents residing in the Region of Murcia and their respective mothers. Interview dyads (adolescent and mother) were incorporated sequentially until data saturation was reached. Transcription and data pre-analysis were conducted simultaneously, following the steps outlined in Interpretative Phenomenological Analysis (IPA). **Results:** The results are structured based on personal barriers of the participants, perceived barriers in interactions with professionals, and institutional barriers to receiving quality healthcare. **Conclusions:** This study helps raise awareness about the situation faced by trans adolescents in healthcare settings. It is essential for healthcare professionals to receive greater training and awareness regarding transgender health and for centers to have action protocols, services, and facilities tailored to these patients.

## 1. Introduction

Transgender people (those whose gender identity differs from the sex assigned at birth) experience discrimination from the general population [1]. This discrimination has a significant impact on their lives and health. The discrepancy between the sex assigned at birth and gender identity can occur as early as childhood and adolescence, and in cases like this, it is very common for adolescents and their families to seek medical advice if they wish to undergo the transition process [2]. Adolescence is a particularly vulnerable stage during which identity is formed and young people prepare for adulthood. For this reason, it is vitally important that healthcare providers offer them quality support and care, as this period is crucial for their development [3]. Trans people suffer violence related to their gender identity, and in many cases, their environment ends up pushing them into ostracism and marginalization, which can sometimes lead to substance abuse or a greater risk of sexually transmitted diseases [4]. Mental health is also a major issue, as anxiety and depression are much more common in this group than in the general population. Factors like discrimination, gender dysphoria, and lack of supportive networks contribute to these health problems. Recent studies estimate that around 40% of the trans population has attempted suicide at least once in their lives [5,6], and an alarming number of trans adolescents have engaged in self-harm. As an example, a study conducted at Boston Children’s Hospital reported a worrying 21% rate of self-harm among participants in its Gender Management Services program, as well as a 9% rate of suicide attempts [7]. This places significant health needs on the shoulders of trans adolescents, in addition to the trans-specific needs associated with the transition process. Therefore, health services play an important role in the well-being of trans people, and counseling for adolescents is vital [8].

Some studies emphasize the vital importance of family support in the lives of trans youth. This parental support is associated with higher self-esteem and significant mental health benefits [9] and may even be considered a protective factor for these adolescents.

### 1.1. Background

Numerous studies have highlighted barriers to healthcare access for transgender people, documenting cases of professionals who harbor prejudices, discriminate against, or fail to adequately treat their patients due to their gender identity [10,11,12]. Although most professionals are well-disposed towards trans people, they often do not receive any specific training during their formation, resulting in physicians having little knowledge and experience regarding the specific needs of these patients [13,14]. For these reasons, in some cases, trans people are reluctant to go to health services for fear of the treatment they may receive, or because of bad experiences in the past [15]. Professionals must be aware of these needs in order to provide personalized, high-quality care to their trans patients, as they may seek healthcare services due to the transition process, in addition to several other reasons. Health professionals must be able to advise the patient and be at their side during the process, which requires appropriate training, which in many cases is not provided [16].

The lack of accessibility to the health system can be detrimental to the well-being of trans patients [17], especially those who are younger who may be exposed to microaggressions and school victimization [18,19]. Some studies carried out in Spain concluded that another major barrier for trans people lies in the lack of training of healthcare personnel and, sometimes, the prejudices they may have towards people from this group [20].

Healthcare for transgender people in Spain is still under development, with substantial differences between the 17 Autonomous Communities. Protocols are still very scarce, having been published in several of these autonomous communities, but not all [21]. Four of these communities have Gender Identity Units (GIUs), where trans patients are referred to receive comprehensive and multidisciplinary care. These units are made up of professionals from various health specialties with specific training to provide trans-competent care, and they use an individualized protocol supported by scientific evidence. However, due to the lack of health services for trans people throughout the Spanish territory, patients often have to be referred to other autonomous communities, and waiting lists are long, especially for those users who wish to access gender-affirming surgery [22].

### 1.2. Gaps and Current Study

Due to the invisibility of this group throughout history, transgender people are still a new topic for the research and healthcare community. In the scientific field, existing studies and articles that address the problems faced by trans people are still very scarce, and many of them focus on problems such as venereal diseases, which are sometimes still considered “typical” of this stigmatized group [23,24]. New studies are slowly emerging on other topics such as the discrimination they suffer, but more evidence is still needed [1].

On the other hand, much of the evidence available to us so far comes from studies conducted in the United States, Canada, and Northern European countries. For this reason, we believe it is necessary to investigate access to the healthcare system for transgender people in Spain, especially those at a vulnerable stage such as adolescence. To do this, we pose the following research question: What was the experience of healthcare access for transgender and gender-diverse adolescents in the Region of Murcia?

The objective of the study was to explore the healthcare access experience of trans and gender-diverse (TGD) adolescents in the Region of Murcia.

## 2. Methodology

To answer the research question posed, an exploratory qualitative study was chosen, adopting an interpretivist paradigm, which assumes that meaning of reality emerges through interaction between the researcher and participants [25]. The study employed an interpretative phenomenological approach (IPA) [26], which aims to “explore the participant’s view of the world and to adopt, as far as is possible, an ‘insider’s perspective’ of the phenomenon under study” [27]. This approach was particularly suitable for exploring how transgender adolescents and their parents experience and interpret their access to healthcare services in a specific region of Spain.

### 2.1. Participants

The participant recruitment process began with researchers contacting associations and advocacy groups for families of TGD adolescents located in the Region of Murcia. The project was presented to the associations, which distributed the information among their members. One participant and their mother were recruited using this method, and the remaining 9 adolescents and their parents were recruited using snowball sampling.

According to the World Health Organization, adolescence is the period of life between childhood and adulthood, between the ages of 10 and 19 (2022). Therefore, the inclusion criteria for the study sample were adolescents between 10 and 18 years old who do not identify with their sex assigned at birth (trans boy, trans girl, and non-binary adolescents) and who resided in the Region of Murcia, Spain, and their families. Parents/guardians were invited to participate in an individual interview. In all cases, it was the mothers who conducted the individual interview.

The study sample comprised 10 adolescents living in the Region of Murcia and their respective mothers, resulting in a total of 20 participants. All participants identified themselves as white. The sample size is appropriate for Interpretative Phenomenological Analysis (IPA), which emphasizes depth over breadth and typically involves small, purposive samples [25]. This number allowed for a rich, idiographic analysis while maintaining analytic depth and manageability.

The adolescent participants ranged in age from 11 to 16 years old (average 14 years old). 7 of them were trans boys and 3 of them were trans girls. Adolescents explain that they became aware that their gender identity differed from the one assigned at birth between the ages of 7 to 13. 5 of the participants were undergoing treatment with puberty blockers at the time of the interview.

Regarding the relatives they lived with, 2 of the 10 participants lived only with their mother and a sibling. Six of them lived with both parents, and one of the cases lived with their mother and her current spouse, who was not the adolescent’s biological father. One of the cases lived with their foster parents and brother. 8 of the 10 participants were enrolled in compulsory secondary education at the time of the study, with the exception of the youngest participants, who were in the final years of primary education. The average grades of most teenagers ranged from passing to excellent, with one case achieving outstanding grades.

Regarding the parents, the participants were women between 32 and 50 years old, mothers of adolescents, with a variety of family types: six married, two divorced, one single mother, and one separated. All of them have at least one other child in addition to the one linked to the study. Seven of them are actively employed. Their educational level ranges from primary education to higher doctoral studies. Two mothers held university degrees in psychosocial disciplines (psychology and social work), while the others, with lower educational attainment, were employed in manual or service occupations. One of them is a shoe store owner. Eight of the mothers use their child’s chosen pronouns, while two continue to use their previous name and pronouns, with the adolescent’s consent. One of the interviewees mentions that her partner makes no effort to adapt to the new family reality. The sociodemographic profile of the participants is shown in Table 1.

### 2.2. Data Collection Procedure and Instruments

Data were collected through in-depth semi-structured interviews designed to elicit detailed first-person accounts of participants’ experiences and meanings in their own words, which facilitated the production of detailed, nuanced narratives essential for idiographic and interpretative analysis. The interviews were conducted in person at locations chosen by the participants: 14 were held in their homes and two in public places (a library cafe). Due to COVID restrictions, the last 4 interviews were conducted by videoconference (zoom platform). In all cases, a semi-structured interview was conducted individually with the adolescents beforehand, followed by an interview with their mothers. The interview questions were open-ended and flexible, encouraging participants to describe events, emotions, and interpretations in their own words. Separate interview guides were developed for adolescents and caregivers to explore key topics such as gender identity, transition processes, and experiences with healthcare services. (see interview guides in Supplementary Materials). A member of a transgender family association collaborated in the development of the guides to ensure that the language used was concise, inclusive, and sensitive to participants’ realities. The semi-structured format allowed questions to be adapted to participants’ responses, fostering a conversational flow and encouraging the sharing of personal experiences. As recommended by Mishler [28], interviews were considered as co-constructed spaces where meanings emerged through interaction between interviewer and interviewee.

All interviews were conducted by one of the researchers (D.M.C), a nurse specializing in bullying among transgender youth and with prior experience in data collection. The author reflected on his own positionality (white, young, cisgender male, nurse, and doctoral student in health sciences) through a reflective journal throughout the process. The rest of the researchers are professionals from different health fields with extensive scientific experience in qualitative methods and sensitive to the topic due to academic or personal experience (being a mother of a gender diverse child) with trans people. Reflexive notes were kept throughout data collection and analysis to monitor assumptions, emotions, and interpretive decisions. Regular discussions with the research team and peer debriefing were used to enhance reflexive awareness and credibility.

Each interview began with a short socio-demographic questionnaire that gathered information on age, sex assigned at birth, gender self-identification, race/ethnicity, family structure, school year, average grade from the previous term, and parents’ occupation and educational level. Interviews with adolescents lasted between 30 and 50 min (average: 35 min), while interviews with mothers ranged from 45 to 90 min (average: 50 min). The interviews were recorded for later transcription. To identify adolescent participants in the transcripts and testimonies, “T” followed by the participant number was used, and “M” followed by the participant number was used for mothers. The testimonies with the same number (example M1 and T1) are mother and child. Prior to the interview, the mothers signed their own informed consent and that of their children, as they were minors, and the adolescents signed their informed assent. Participants also gave their consent to have their interviews recorded using a voice recorder. The study has been approved by the Ethics Committee of the University of Murcia under code CI2772 (approval date: 29 April 2020).

### 2.3. Analysis

The audio of the interviews was transcribed verbatim by the researchers. Fieldnotes made during the interviews were also added to the transcripts. Transcriptions and data pre-analysis were carried out simultaneously.

Interview dyads (adolescent and mother) were incorporated sequentially until no substantially new codes or meanings emerged, which was discussed within the research team after the eighth participant dyad. Two additional dyads were then included to confirm the stability and comprehensiveness of the themes.

Data analysis followed the steps outlined in Interpretative Phenomenological Analysis [27]. Each transcript was read several times to achieve immersion, and exploratory comments were made focusing on descriptive, linguistic, and conceptual aspects.

Meaning units were then identified and transformed into emergent themes. Pairwise coding between two researchers (MMR and MPB) was carried out, and the data were analyzed inductively, incorporating emerging and in vivo codes. Consensus was reached through discussion when discrepancies arose. Connections across themes were explored to develop higher-order categories, while constantly referring back to the original transcripts to ensure that interpretations remained grounded in participants’ words. Atlas.ti 8.4 was used to organize and retrieve coded segments. Ultimately, the analysis yielded six subcategories, which were then clustered into three overarching categories representing the core dimensions of participants’ lived experiences (see Table 2).

Reflexivity was also maintained throughout data analysis. Reflexive journaling and regular team discussions helped to balance sensitivity with analytic distance. Interpretations were continuously examined in light of the researcher’s positionality to ensure that themes reflected participants’ meanings rather than preconceived assumptions.

Given that interviews were conducted separately with adolescents and their mothers, a dyadic analytic lens was applied to examine convergence and divergence across their narratives [29]. Initial coding was performed for each group independently to preserve the integrity of their lived experiences and then comparatively to explore points of convergence, divergence, and complementarity. Divergent accounts were treated as expressions of the relational and contextual complexity surrounding healthcare access.

The rigor of the study was ensured by adhering to the criteria of credibility, transferability, reliability, and confirmability [30]. Credibility was reinforced through detailed field notes documenting contextual and nonverbal cues, verbatim transcription of interviews, and supervision by an expert in qualitative methodology to ensure the quality of the analysis and reduce potential biases [31]. The first author maintained a reflective journal throughout the process to examine their positioning and monitor their own assumptions and interpretations. Peer review and discussions with the research team fostered interpretive consistency. Transferability was supported by purposive sampling and a contextualized description of the participants and their experiences [32]. Reliability and confirmability were ensured through an audit log documenting analytical decisions and the evolution of codes, as well as through consensus among co-authors in the development of emerging themes. This dyadic comparison enriched interpretation and enhanced the credibility of findings.

## 3. Results

Interviews with adolescents and their mothers yielded extensive information about trans adolescents’ experiences with healthcare and barriers to receiving healthcare. Results were organized into three main categories: (I) Personal barriers of the adolescent, (II) Perceived barriers in interactions with professionals and (III) Institutional barriers. These barriers were interrelated across different levels of influence: micro (individual and interpersonal experiences), meso (organizational structures and practices), and macro (policy and systemic frameworks). Some professional barriers were understood as spanning both micro and meso levels, reflecting how individual encounters are shaped by institutional dynamics.

### 3.1. Personal Barriers of the Adolescent

At the micro level, adolescents described fear of discrimination when seeking healthcare. Some expressed shame and rejection of having their pre-transition name used because they had not yet been able to change their name on their ID cards.

*Because before I didn’t have an ID, so they didn’t call me by my name [...] They called me by the other one and I was embarrassed*.

(T1)

*It bothered me, I wanted to tell them that it wasn’t my name […] I don’t like being called by the other name*.

(T7)

The use of a pre-transition name can undermine adolescents’ affirmed identities, reinforcing feelings of invisibility and lack of recognition within institutional contexts. Likewise, mothers reported noticing their children’s shame and fear when faced with situations where they might be called by their old name or where they might have to explain their gender identity to both healthcare providers and other patients, so they displayed a protective attitude to avoid awkward situations for their children.

*If she went alone, it would be harder for her, but since I’m always the one who goes, I’m the one who’s always there for her. It’s like a constant struggle, and I often say, “If I could just change her name, we’d be spared these situations”*.

(M10)

*It’s true that people look. They used to look, more than now. But they looked, and then I looked them straight in the eyes, as if to say, “If you don’t look away, I’ll explain it to you”*.

(M7)

These narratives reveal a dyadic dynamic in which mothers act as emotional and social buffers, mitigating potential exposure to stigma while managing their own anxiety about how their children might be perceived.

One participant expressed fear of being denied hormone treatment. In contrast, participant T5 expressed fear of being included in the transition program without having received sufficient information and made a thoughtful decision about it:

*“I’m afraid they’ll put me in the transition program […] I’d like to learn a lot more before embarking on that process”*.

### 3.2. Perceived Barriers in Interactions with Professionals

In the area of healthcare professionals, at both micro and meso levels, adolescents and mothers described barriers related to the lack of training among healthcare professionals on transgender issues, as well as the attitudes of these professionals themselves toward this group.

### 3.3. Perceived Training of Professionals

Perceptions of professional training varied across participants and between dyads. At the meso level, most adolescents considered that the healthcare professionals who treated them were sufficiently trained and provided them with appropriate care.

*I go to my nurse to get the shot and I don’t feel any discrimination, nor do I feel like she looks at me badly... and when I ask her, I know that she can give me a good answer, that she has information on the subject of hormones, testosterone and all that*.

(T4)

While some of them expressed their perception that research and training for professionals in trans-specific care matters is insufficient, they identified the need for specialized professionals in this area.

*The work on transition and all that isn’t given much importance, so there aren’t many advances either [...] There should be more doctors who are more specialized, not just any psychologist, or an endocrinologist who generally works on the whole body. But someone who works specifically on that topic*.

(T3)

This sense of professional insufficiency was echoed by mothers, some of whom appreciated the dedication and empathy of the clinicians they encountered. However, opinions differed depending on the specific professional involved. Some mothers believe that the professionals are well-trained and knowledgeable about transgender-related issues, expressing overall satisfaction with the care received.

*“Apart from the psychological aspect, which needs improvement, the rest of the staff is super super prepared, and also at a level... The endocrinologist explained things to us very well and we asked twice because we didn’t understand the first time and were embarrassed the second time, and he told us to ask as many times as necessary until we understood”*.

(M6)

Others, however, reported the opposite experience, emphasizing the lack of knowledge about the trans community and trans-specific care.

*Even the most basic concepts aren’t clear. Starting with the example of my pediatrician, [...] she says she’d never had a case, never seen anything, and knew absolutely nothing, not even where to refer someone or anything like that; well, I think there are a lot of people in the healthcare field who don’t have even the basic training”*.

(M1)

### 3.4. Perceived Attitude of Professionals

Adolescents and their mothers described contrasting experiences in their encounters with healthcare professionals, revealing how attitudes toward transgender and gender-diverse patients operate at the micro level. recalled moments in which professionals’ words or gestures made them feel questioned and judged, evoking discomfort and mistrust.

*You tell them something and they always question you, and... that’s quite annoying […] She addressed me as a man, even though I had said it and even though she knew it, because I think she specializes in these cases, with trans people. So... it seems a bit unprofessional to me*.

(T6)

*When they came to ask me my name, they thought I was a boy, and when I came in, they told me I was a girl. It was a little strange, a little rude*.

(T10)

These experiences reflect how even subtle forms of misrecognition or doubt disrupt the adolescents’ sense of legitimacy and self-determination. The interactional tension between professional authority and the adolescents’ self-knowledge highlights the asymmetry of power between professionals and adolescents.

In the case of mothers, on the one hand, we find positive experiences, in which they have not felt discrimination or rejection by health professionals, which they can consider a blessing, as expressed by participant M8:

*“We’ve been very lucky. Well, it’s not exactly luck, I think it’s what it should be, but the truth is that we’ve been with professionals who have listened to us and treated us very well”*.

They also identified a lack of naturalness in addressing issues related to transition or trans-specific care.

*Just see it as something natural. It’s not that they’re doing a bad job, because they’re not doing it badly, but if, for example, someone comes along who turns out to be named Margarita and then turns out to be José, they should see him as a normal person, that’s what I mean*.

(M10)

### 3.5. Institutional Barriers

Participants also identified several institutional barriers situated at the meso (organizational) and macro (structural and policy) levels, all contributed to experiences of exclusion and invisibility. One of these is spatial resources, referring to the rooms and restrooms in hospitals and healthcare centers themselves, such as gender-segregated rooms and restrooms. The second main barrier is the absence of protocols for trans patients within healthcare institutions. The last barrier identified at the institutional level concerned administrative issues related to name and gender changes in health records.

### 3.6. Spatial Resources

Beyond interpersonal encounters, participants’ experiences were also shaped by the material and spatial organization of healthcare settings (meso-level). Spatial resources, such as hospital rooms and gender-segregated restrooms, became symbolic sites of inclusion or exclusion. One of the mothers commented that, despite the good treatment her son received from professionals, he was assigned to a gendered hospital room that did not correspond to his gender identity:

*My son was treated wonderfully when we went to the emergency room. Even the cleaning lady addressed him using male pronouns. But where did they put him? In a room with a girl*.

(M1)

Some adolescents expressed concern about using gender-segregated restrooms in hospitals or health centers, especially those whose gender identity is not recognized. One of the alternatives they proposed was the provision of unisex restrooms.

*I would like there to be a little more diversity and that... although it may seem strange, that boys were in the same bathroom as girls*.

(T2)

*I don’t have any problems because I see myself as a woman and people see me as a woman and I can go into the girls’ restroom, [...] but I also understand that there may be people who think the opposite, who prefer the restrooms to be mixed*.

(T9)

Mothers’ views on this issue were more diverse. Some perceived it as minor compared to other structural barriers, so they didn’t give much importance to the issue. While others explicitly supported unisex restrooms, recognizing the emotional relief they provided to their children:

*(My son) always complains a lot about the restrooms. He feels very comfortable when he goes somewhere and sees that there are unisex restrooms. In that regard, he always says so, and yes, the truth is, we would prefer it*.

(M8)

### 3.7. Lack of Protocols and Coordination

At the meso and macro levels, mothers consistently described the absence of a clear pathway. They expressed the need for an established protocol for the entire region, as they feel disadvantaged by the lack of knowledge and, in particular, the lack of coordination among professionals and institutions.

*Coordination is needed, and a protocol is needed. In fact, one is being created, but in the meantime, the staff is confused. The primary care physician doesn’t know if they should refer the patient directly to the endocrinologist; sometimes they refer them to mental health; they don’t know that it’s no longer necessary to go to mental health, because it has become depathologized. So, from mental health, they sometimes send the patient back to the primary care physician, then the primary care physician refers them to the endocrinologist. […] This applies to the healthcare field and then to the administrative field as well*.

(M1)

Other mothers illustrated how bureaucratic detours and lack of coordination prolonged referral chains that delayed access to specialized care, reflecting systemic failures in coordination.

*With the pediatrician, it took a year… First they send you to the psychiatrist, from the psychiatrist they refer you back to the pediatrician, second visit, and then they send you to the endocrinologist*.

(M4)

### 3.8. Health Records

At the macro level, participants described administrative and information-system barriers related to the registration of gender and name changes. Due the persistence of incongruent information across databases, the mothers expressed the need for a health registry documenting the name and gender of trans people, as the name change process may not yet have been completed on their National Identity Document. This would prevent adolescents from being called by their previous name, avoid confusion among professionals, and provide unity for this community.

*When we decided on her new name, we wanted the Social Security system to not use the ID card or the family book, but rather to allow you to create a document that doesn’t require doctors to be constantly correcting the old name*.

(M3)

*His real name hadn’t been changed yet. He had to be admitted to the (referral hospital). We went through the ER, and there was confusion in the sense that when he came through the ER, the papers indicated one thing, and the medical record indicated another. When I approached him, I said Daniel. His appearance didn’t match, even the doctor hesitated. “Are you so-and-so?” because the name was feminine. Because he didn’t know if he was seeing the right patient with the right report, so all those things are important to fix as soon as possible [...]. And then it does generate bad situations whenever the documentation isn’t changed. That’s why we are in such a hurry to get the laws changed*.

(M2)

## 4. Discussion

This study aims to answer the research question posed: What are the experiences of healthcare access for transgender and gender-diverse adolescents in the Region of Murcia? This study also identifies the barriers that TGD adolescents may experience when receiving healthcare. These barriers can be divided into institutional barriers, perceived barriers in interactions with healthcare professionals, and personal barriers imposed by the trans users themselves. The findings of this study illustrate that barriers to healthcare for TGD adolescents operate across interconnected system levels.

At the micro level, barriers emerged from personal experiences and interpersonal interactions between adolescents, caregivers, and healthcare professionals. Although most participants considered that they received good care from the healthcare professionals they dealt with, the main personal barrier that adolescents encountered was the fear of rejection or discrimination during healthcare, both from healthcare professionals and from other users. This has been described by Tutić Grokša et al. [33] as an anticipation barrier.

The stigma and discrimination experienced by trans people in healthcare has been documented internationally [34,35], leaving well-being and healthcare access as privileges for some, in which TGD people are excluded through various barriers [36]. In response to anticipated discrimination, participants engaged in self-protective strategies, such as seeking care only from familiar professionals, selectively disclosing or concealing their gender identity to avoid having to give explanations or uncomfortable questions, and avoiding going to the health system, which is also evident in the Transgender Europe report [37].

Regarding barriers from health professionals, although various international studies show a growing acceptance of health professionals towards LGBT people, there are still cases of discrimination by professionals towards these patients, especially TGD people [38,39], which may be a reflection of society and university communities [40].

Some participants reported situations in which healthcare professionals addressed them by the wrong pronoun or questioned their gender identity, making them feel judged, as also described by Strauss et al. [41]. This may be due to a lack of training and reluctance of asking trans and gender-diverse patients for fear of being perceived as disrespectful [42]. Such professional discomfort can inadvertently reinforce barriers to gender-affirming healthcare.

This barrier must be resolved as soon as possible, since it has been shown that good treatment by health professionals has a beneficial effect on the mental health of trans patients [43]. On the other hand, several participants agreed that healthcare professionals need more specific training on TGD health, especially in the areas of psychology and hormone treatment. Training in the specific health needs of these patients is still very limited [44]. These findings highlight that professional barriers are rooted in meso-level organizational deficits including insufficient education, supervision, and institutional accountability for gender-inclusive practice. However, ways of including material on TGD people in the curricula of foreign [45] and Spanish [46] universities are gradually being considered, yielding promising results.

Finally, we encounter barriers related to the healthcare institutions themselves, especially those with single-sex facilities, such as shared rooms or restrooms. Some participants expressed a certain degree of discomfort with having to use these restrooms, as they are exposed to the judgment of other users. One option mentioned by some participants is to include more diverse facilities, such as unisex restrooms. There have been instances of some policies that called for transgender people to use restrooms of the gender they desired, although unisex restrooms are also considered a viable option [47]. However, while this can contribute to trans people’s safety and privacy, it can also mean excluding them from using their preferred gender’s restrooms. Trans-exclusionary groups have frequently advocated against the inclusion of trans people in these public facilities, and it remains a controversial topic in the community [48].

Similarly, within institutional barriers, we also find the absence of action protocols aimed at treating TGD patients. The diversity of healthcare systems within the country results in differences in healthcare access. Recently, the Murcian Health System published a Regional Protocol for Healthcare for Transgender People [49], although its effects have yet to be assessed. The protocol reflects a depathologizing approach to transgender healthcare and removes the requirement for a psychological assessment to access trans-specific services. Under this system, patients first consult their primary care physician, who then refers them to the appropriate specialist. This pathway applies to services such as hormone therapy, puberty suppression, mental health support when needed, and gender-affirming surgery, although patients may still encounter gatekeeping practices and long waiting lists before accessing these services. Another institutional factor that generates anxiety and fear is the administrative bureaucracy involved in changing one’s name and gender marker in the health registry. Being called by their previous name often causes discomfort and the perception of being judged by both healthcare professionals and other users of the health system [50]. This lack of social support increases levels of anxiety and depressive symptoms [51].

Furthermore, TGD patients encounter healthcare biases and systemic barriers related to protocols and electronic health record systems that are still organized around binary notions of biological sex. This can result in the exclusion of trans men from cervical cancer screening programs or the omission of prostate examinations for trans women [52,53]. These macro-level barriers, in turn, shape experiences of exclusion, frustration, and mistrust of healthcare institutions among TGD people, and highlight the need for comprehensive system reform.

Taken together, these findings demonstrate that improving healthcare access for TGD adolescents requires coordinated action across micro, meso, and macro levels. Improving the lived experience of TGD adolescents in healthcare involves not only transforming professional practices but also reconfiguring the institutional and policy frameworks that result in exclusion. A multi-level approach is therefore essential to transform both everyday interactions and the structural conditions that shape them.

### Study Limitations

Among the limitations of the study is the age of the participants, which may limit the information provided, either due to their ability to express themselves or because they may find a face-to-face interview intimidating. To counteract this potential intimidation, the children were accompanied by their father or mother to the interview, and an introduction to the interviewer and the interview took place in a friendly setting chosen by the participants. Information was triangulated by also conducting in-depth interviews with the parents. Although both parents were given the option to conduct the interview, it was always the mothers who volunteered, which may limit the range of caregiver perspectives. This may reflect their greater involvement and support for their children, although this remains a topic for further exploration.

Recruitment of participants was conducted through trans associations and snowballing. This may represent a bias towards families who are already involved in the community and exclude trans adolescents who lack support from their families or are disconnected from supportive networks.

Participants share their experiences of accessing the healthcare system in the Region of Murcia, whose healthcare protocol for trans people is recent and was published after the interviews were conducted. Consequently, their accounts reflect a context prior to the full implementation of the depathologizing model. Experiences may differ in autonomous communities where such protocols have been in place for a longer period or in regions that still lack a specific regulatory framework. Although these findings are context-specific to the Region of Murcia, several identified mechanisms (such as variability in professional attitudes, administrative and electronic record rigidity or prolonged waiting lists) reflect patterns observed across Spain’s decentralized healthcare system. Therefore, the study’s contribution lies in its theoretical transferability, highlighting structural and relational mechanisms that may inform policy and practice in other autonomous communities with similar contexts.

## 5. Conclusions

The study concludes that trans adolescents encounter various barriers in accessing healthcare services, whether personal, professional, or institutional, which mutually reinforce each other. This study can contribute to raising awareness of the situation these adolescents face in an environment that should be supportive of them, as they are at a stage where adequate care is vital for their development. Based on these findings, we propose several actionable strategies at different levels:

Micro (professional): Strengthen training in gender-affirming communication, inclusive language, and care practices that respect diverse anatomies.

Meso (organizational): Establish liaison roles or designated reference professionals within health centers to coordinate referrals and reduce waiting times.

Macro (policy): Ensure consistent implementation of the regional protocol, allocate sufficient resources for trans-specific services, adapt facilities to the needs of transgender and gender-diverse users, include non-binary gender markers in electronic health records and develop inclusive health promotion campaigns to reduce anticipatory fear of discrimination.

These actions could mitigate many of the service-level barriers identified and foster a more inclusive and coordinated system of care.

Further research is needed to assess how the progressive implementation of regional transgender healthcare protocols and policies (macro-level) influences access experiences among TGD adolescents and their families over time, as well as how regional mechanisms mediate these processes.

## Figures and Tables

**Table 1:** Sociodemographic profile.

Adolescents	Variable	*n* (%)
	**Age**	
	11	2 (20%)
	12	1 (10%)
	14	1 (10%)
	15	3 (30%)
	16	3 (30%)
	**Gender identity**	
	Boy/Male	7 (70%)
	Girl/Female	3 (30%)
	**School year**	
	Primary Education	2 (20%)
	Compulsory Secondary Education	7 (70%)
	1st year of high school	1 (10%)
	**Environment**	
	Urban	5 (50%)
	Rural	5 (50%)
**Parents**	**Marital status of mothers**	
	Married	7 (70%)
	Divorced	2 (20%)
	Single	1 (10%)
	**Educational level of mothers**	
	Primary Education	3 (30%)
	Secondary Education	2 (20%)
	Higher Education	1 (10%)
	Vocational Studies	2 (20%)
	University Studies	2 (20%)
	**Employment status of mothers**	
	Employed	7 (70%)
	Unemployed	3 (30%)

**Table 2:** Categories and subcategories in data analysis.

Categories	Subcategories
Personal barriers of the adolescent	-Fear of discrimination
Perceived barriers in interactions with professionals	-Perceived training of professionals -Perceived attitude of professionals
Institutional barriers	-Spatial resources -Lack of protocols and coordination -Health records

## Data Availability

The original contributions presented in this study are included in the article. Further inquiries can be directed to the corresponding author.

## Supplementary Materials

The following supporting information can be downloaded at: https://www.mdpi.com/article/10.3390/healthcare13222953/s1, File S1: Interview guides.
