# Supplementary material for: Experiences of Health Care Access Among Trans Adolescents in the Region of Murcia, Spain
Source: Healthcare (Basel). 2025 Nov 18;13(22):2953. doi: 10.3390/healthcare13222953 (PMC12652444; doi:10.3390/healthcare13222953)
Supplement: Supplementary file 1 [file healthcare-13-02953-s001.zip › healthcare-3948181-supplementary.pdf]

Interview protocol for adolescents.

Sociodemographic data
- Age
- Sex assigned at birth
- Family structure (people they live with)
- School year
- Average grade for the last term
- Educational level of parents
- Environment (urban/rural)
- Ethnicity

In-depth interview

To begin: how would you like me to address you?

-How would you describe yourself? (Gender identity).

-When did you begin to realize your gender identity?

-Are you undergoing any transition process? (Hormones, clothing). What do you think of this process?

-Who are the people who support you the most regarding your gender identity?

-Have you had negative experiences related to your gender identity? In that case, could you describe a time when you have experienced any kind of harassment due to your gender identity? (Insults, gestures, emails, chats, text messages).

-How was your experience accessing healthcare related to your trans-specific needs?

-What feelings has this healthcare experience generated in you?

-What was your experience regarding other users in health services?

-How were you treated by healthcare professionals?

-How would you like the care you receive from health professionals to be?

-To what extent do you think the facilities in hospitals/health centers are suitable for you? (gender-separated rooms and restrooms)

-Is there anything in the healthcare system you would like to change?

Interview protocol for caregivers

Sociodemographic data
- Age
- Marital status
- Family structure (people they live with)
- Number of children

- Educational level
- Employment status
- Profession
- Environment (urban/rural)
- Ethnicity

In-depth interview

-How would you like me to address your child?

-What is your child's felt gender identity?

-At what point did you realize that your child's gender identity is different from the one assigned at birth?

-What was your reaction? What feelings do you remember most intensely about the aforementioned period?

-To what extent have you had any professional advice (from doctors, psychologists, therapists, or other professionals) on how best to address your child's concerns and behaviours?

-Do you think your family's socioeconomic status can positively or negatively influence the care your child receives? Why?

-How was your child’s experience accessing healthcare related to their trans-specific needs?

-What feelings has this healthcare experience generated in you?

-What was your experience regarding other users in health services?

-How were you and your child treated by healthcare professionals?

-Regarding healthcare professionals, how would you like the care your child receives to be?

-To what extent do you think the facilities in hospitals/health centers are suitable for your child?

-What would you like to change about the healthcare you and your child have received?
